# Accelerometer-measured physical activity and risk of incident ulcerative colitis: a population-based prospective study

**DOI:** 10.3389/fpubh.2026.1955306

**Published:** 2026-09-22

**Authors:** Zitong Li, Haoran Ke, Hanxiao Feng, Qianyun Lin, Ye Chen

**Affiliations:** 1 Integrative Clinical Microecology Center, Shenzhen Key Laboratory of Gastrointestinal Microbiota and Disease, Shenzhen Clinical Research Center for Digestive Disease, Shenzhen Technology Research Center of Gut Microbiota Transplantation, Department of Gastroenterology, Shenzhen Hospital, Southern Medical University, Shenzhen, China; 2 Department of Gastroenterology, The First Affiliated Hospital of Shantou University Medical College, Shantou, China; 3 Hepatology Unit, Department of Infectious Diseases, Nanfang Hospital, Southern Medical University, Guangzhou, China; 4 Department of Gastroenterology, State Key Laboratory of Organ Failure Research, Guangdong Provincial Key Laboratory of Gastroenterology, Nanfang Hospital, Southern Medical University, Guangzhou, China; 5 Department of Gastroenterology, Beijing Friendship Hospital, Capital Medical University, Beijing, China

**Keywords:** accelerometer, IBD, physical activity, primary prevention, ulcerative colitis

## Abstract

**Background:**

Ulcerative colitis (UC) lacks established primary prevention strategies despite its economic burden. Physical activity (PA) is promising, yet studies using self-reported data show inconsistent results. We examined the association between accelerometer-measured physical activity and UC risk in a large prospective cohort.

**Methods:**

This cohort study analyzed data from 95,197 UK Biobank participants without baseline inflammatory bowel disease and with valid accelerometer data, with follow-up from 2013 to 2015 through 2023. Machine learning models estimated time spent in sedentary behavior, light physical activity (LPA), and moderate-to-vigorous physical activity (MVPA). MVPA patterns were categorized as active weekend warriors (≥231.5 min/week, ≥50% in 1–2 days), active regular (≥231.5 min/week, not meeting weekend warrior criteria), and inactive (<231.5 min/week). Cox proportional hazards regression assessed associations between PA and UC risk.

**Results:**

Over 8.86 years median follow-up, 247 incident UC cases were recorded. MVPA showed a dose–response relationship with UC risk (trend *p* = 0.004). Compared to the lowest MVPA quartile, the third (231.5 to <402.3 min/week) and highest (>402.3 min/week) quartiles showed multivariable adjusted hazard ratios (aHRs) of 0.59 and 0.63 (95% CI: 0.40–0.85 and 0.43–0.91). Neither LPA nor sedentary time showed significant associations with UC risk. Both weekend warrior (aHR: 0.72, 95% CI: 0.53–0.99) and regular MVPA patterns (aHR: 0.57, 95% CI: 0.39–0.82) correlated with reduced UC risk. These associations remained robust across various subgroups and sensitivity analyses.

**Conclusion:**

Accelerometer-measured MVPA is associated with reduced UC risk. Both weekend warrior and regular MVPA patterns conferred protection, suggesting that flexibility in exercise scheduling may be feasible.

## 1 Introduction

Ulcerative colitis (UC), a lifelong inflammatory bowel disease (IBD), poses a growing global health challenge (1–3). With a worldwide prevalence of 5 million cases as of 2023 (1) and rising incidence rates in newly industrialized and select high-income nations (2, 3), UC imposes substantial economic burdens on healthcare systems (2, 3). The absence of established preventive strategies underscores the critical need for research into potential risk-reduction measures.

The etiology of UC involves both genetic and environmental factors, including sedentary lifestyle (4). Physical activity (PA), the energy-expending movement of skeletal muscles (5), has shown beneficial effects on various non-communicable diseases (6). Recent guidelines encourage regular exercise for IBD patients (7). However, Prospective studies on the PA-UC risk relationship have produced mixed findings. The Nurses’ Health Study, involving 194,711 women, found no significant link between high PA levels and UC risk (8). Intriguingly, a follow-up analysis of the same cohort suggested that 42% of UC cases might be preventable through a healthy lifestyle, with adequate PA as a key component (9). Conversely, the European Prospective Investigation into Cancer and Nutrition (EPIC) and a Danish cohort study reported no relationship between PA levels and IBD risk (10, 11). Notably, questionnaire-based PA assessments in these studies face limitations in accurately capturing intensity, duration, and energy expenditure (12), while being prone to recall bias and measurement errors.

While prior studies largely relied on self-reported physical activity (PA) with inherent recall and misclassification biases (13, 14), this study leverages wrist-worn accelerometry in approximately 90,000 UK Biobank participants to objectively quantify the PA-ulcerative colitis (UC) association. Specifically, we aimed to: (1) delineate the precise dose–response relationship and identify a reproducible MVPA threshold (i.e., 231.5 min/week) for UC primary prevention; and (2) compare the protective efficacy of the “weekend warrior” pattern (concentrated 1–2 days) against regular daily MVPA, thereby assessing the feasibility of flexible exercise scheduling. Additionally, we evaluated the independent effects of light PA and sedentary time, and tested the robustness of our findings across diverse subgroups. By providing these accelerometer-derived, quantifiable targets and pattern-specific risk estimates, this study moves beyond methodological refinement to offer evidence-based, temporally flexible strategies for UC prevention, potentially alleviating the growing burden of this disease.

## 2 Methods

### 2.1 Data source and study design

Drawing from the UK Biobank, a large-scale prospective cohort study, this research analyzed data from about 500,000 participants. Recruitment occurred between 2006 and 2010, encompassing individuals aged 40–69 from 22 assessment centers across England, Wales, and Scotland (15). Ethical approval for the UK Biobank study was granted by the North West Research Ethics Committee, with all participants providing written informed consent. The study design adhered to STROBE guidelines for cohort studies. Participants completed a comprehensive baseline assessment, including a touch-screen questionnaire and computer-assisted interview, capturing detailed sociodemographic, lifestyle, and health data. Physical examinations were conducted, and biospecimens were collected.

The analysis focused on a subsample of 103,662 participants who received random invitations via email to wear wrist-worn accelerometers (AX3; Axivity Ltd) continuously for 7 days between 2013 and 2015 (16, 17). The participation rate was 45%, with no significant baseline differences between respondents and non-respondents (17). Accelerometers captured triaxial movement at 100 Hz, with a sensitivity range of ± 8 g. Data were processed following established protocols (17), including gravity calibration. The raw data were condensed into 5-s intervals and quantified using average vector magnitude. Non-wear periods (stationary intervals exceeding 60 min with standard deviation below 13.0 milli-gravity across all axes (17)) were imputed using the average vector magnitude and intensity distribution from corresponding time points on other days.

Exclusion criteria were applied to participants failing accelerometer data quality control (*n* = 7,052), including insufficient wearing time (*n* = 6,984), calibration failure (*n* = 17), insufficient data (*n* = 38), and implausibly high acceleration values (*n* = 13). Participants with prevalent inflammatory bowel disease before wearing the accelerometer were also excluded (*n* = 1,433). The final analytic sample comprised 95,197 participants (Figure 1).

**Figure 1:**
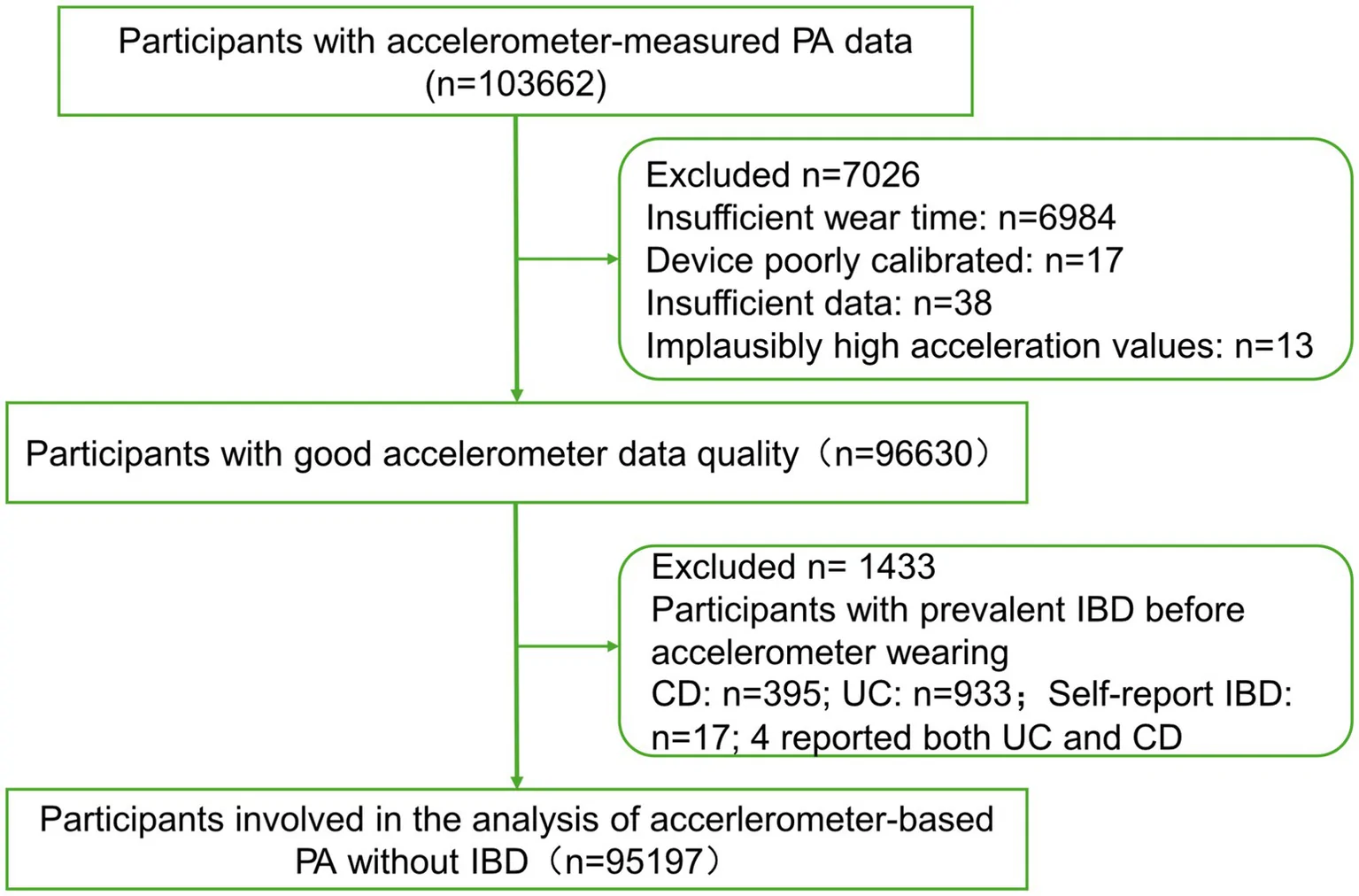
The flow chart of participants through the study.

### 2.2 Exposure assessment

Total mean PA volume was calculated as the average vector magnitude in milligravity units (17). PA intensities were categorized using machine learning models (16) trained on free-living data from time-use diaries and wearable cameras. PA was categorized based on metabolic equivalent of tasks (METs): sedentary time (≤1.5 METs, e.g., driving or TV watching), light physical activity (LPA) (<3 METs, excluding sedentary activities, e.g., slow walking or self-care), and moderate-to-vigorous physical activity (MVPA) (≥3 METs, e.g., swimming, jogging, or cycling). For each participant, total wear duration and time allocated to different activity intensities were computed.

The MVPA proportions were converted to minutes per day, facilitating participant categorization into Inactive, Active-Weekend Warrior, and Active-Regular groups. The primary analysis employed two thresholds: the guideline-based ≥150 min/week (5, 18) and the sample median of ≥230.4 min/week, reflecting UK national health surveys indicating approximately half of the population as physically active (19). Secondary analyses explored additional thresholds. Participants were categorized as active weekend warriors if they met or exceeded the MVPA threshold with at least half of their total MVPA occurring over 1–2 days (6). Those meeting the MVPA threshold without the weekend warrior pattern were classified as active regular, while those below the threshold were deemed inactive.

The UK Biobank Data Showcase provides access to all study variables for registered researchers (Overall acceleration average: Field 90,012, PA intensities: Fields 40,047–40,049, Moderate-Vigorous Day average: Field 40,045).

### 2.3 Ascertainment of UC

The IBD cases were identified using 3-digit ICD-10 code (K51 for UC, K50 for CD) and UK Biobank self-reported data (IBD: Field 1,461, CD: Field 1,462, UC: Field 1,463). Participant hospitalization data were updated until October 1, 2023. Participants were followed until UC diagnosis, death, or the end of follow-up, whichever occurred first; those without UC were censored at their last follow-up date.

### 2.4 Covariates assessment

Potential confounding factors were assessed using data from touch-screen questionnaires, anthropometry, biochemical markers, accelerometer outputs, and medical histories. Confounders examined included sex, age, Townsend deprivation index (TDI), education level, Body mass index (BMI), alcohol consumption frequency, smoking status, non-steroidal anti-inflammatory drug (NSAID) and antibiotic use, and pre-existing cardiovascular disease (CVD), diabetes, and hypertension (identified by self-report or health records pre-accelerometer). NSAID use was included as a covariate given its previously reported potential association with UC risk (9, 20). Additional variables for females were hormone therapy, oral contraception, menopausal status, and parity. All covariates were derived from the baseline assessment (Instance 0, 2006–2010), as it provided complete data for all participants; Instance 1 (2012–2013) was not used due to its limited sample size (*n* = 20,312).

### 2.5 Statistical analysis

Participant characteristics at study entry were summarized by total PA volume quartiles, presenting means with SDs for continuous measures and percentages for categorical variables.

Cox proportional hazards regression models assessed the association between exposure quartiles (sedentary time, LPA, and MVPA) and UC incidence risk. Follow-up started at accelerometer wear completion. Models were adjusted for sex, age, BMI, alcohol consumption, smoking status, socioeconomic status, education level, medical history (including cardiovascular disease, hypertension, and diabetes), medication use (NSAIDs and antibiotics), and female-specific variables (menopausal status, parity, contraceptive use, and hormone replacement therapy). For female-specific variables, we used interaction terms with sex.

Missing covariate data were handled via complete-case analysis, yielding 94,676 participants with complete data for all covariates (99.5% of the original cohort) in the final multivariate models. Supplementary Table 1 details the missing covariates among participants. To evaluate independent associations, we additionally constructed a mutually adjusted Cox model that simultaneously included LPA, MVPA, and sedentary time as quartile-based exposures, with results presented in Supplementary Table 2. Schoenfeld residuals analysis confirmed the proportional hazards assumption, showing no violations. A correlation matrix was generated to evaluate potential collinearity between covariates. No substantial multicollinearity issues were identified, except for the expected high correlations between sex variables and their interactions with female-specific variables.

Restricted cubic spline Cox models evaluated the dose–response relationship between PA, sedentary time, and UC incidence risk. Subgroup analyses assessed robustness and explored variations by stratifying for sex, age (<65, ≥65), BMI (<25, ≥25), TDI (< median, ≥ median), smoking status (never, current or former), or cardiovascular disease status (yes, no). Potential interactions between these risk factors and PA were assessed using cross-classification. Interaction significance was assessed via log-likelihood ratio tests, comparing models with cross-classified categories against those treating factors independently.

Robustness checks were performed to validate the findings. These included excluding events within 2 years post-accelerometer wear to mitigate reverse causality concerns, and employing competing risk models using Fine-Gray subdistribution hazards to assess potential bias, with death as a competing risk for UC incidence.

Analyses were conducted in R 4.4.1, employing two-sided tests with significance threshold *p* < 0.05.

## 3 Results

### 3.1 Characteristics of the study population

The study included 95,197 participants (56.3% women) with a mean age of 62.31 years (SD 7.85) and BMI of 26.7 kg/m^2^ (SD 4.5). Average weekly PA durations were: LPA 2121.90 min (SD 690.21), MVPA 290.06 min (SD 243.30), and sedentary behavior 3949.80 min (SD 763.81) (Figure 1 and Table 1). After excluding participants with missing covariate data, a total of 94,676 participants (99.5% of the original cohort) with complete data were included in the final multivariate regression analyses.

**Table 1:** Participant characteristics by fourths of total mean accelerometer-measured physical activity volume levels.

Characteristics	Overall	Q1	Q2	Q3	Q4
*n*	95,197	23,824	23,821	23,772	23,780
Sex					
Female	53,632 (56.3)	11,896 (49.9)	13,550 (56.9)	14,049 (59.1)	14,137 (59.4)
Male	41,565 (43.7)	11,928 (50.1)	10,271 (43.1)	9,723 (40.9)	9,643 (40.6)
Age at accelerometer assessment	62.31 (7.85)	65.07 (7.31)	63.08 (7.63)	61.58 (7.73)	59.50 (7.65)
BMI (kg/m^2^)	26.71 (4.53)	28.45 (5.15)	26.96 (4.40)	26.25 (4.09)	25.17 (3.70)
Townsend Index score^a^	−1.73 (2.82)	−1.59 (2.90)	−1.78 (2.80)	−1.81 (2.77)	−1.74 (2.80)
College degree or equivalent	42,471 (44.6)	9,820 (41.2)	10,782 (45.3)	10,856 (45.7)	11,013 (46.3)
Cigarette smoking					
Never	54,326 (57.1)	12,613 (52.9)	13,565 (56.9)	13,908 (58.5)	14,240 (59.9)
Previous	34,248 (36.0)	9,071 (38.1)	8,649 (36.3)	8,387 (35.3)	8,141 (34.2)
Current	6,623 (7.0)	2,140 (9.0)	1,607 (6.7)	1,477 (6.2)	1,399 (5.9)
Alcohol consumption					
Never	5,381 (5.7)	1,657 (7.0)	1,277 (5.4)	1,215 (5.1)	1,232 (5.2)
<1 time/week	19,350 (20.3)	5,660 (23.8)	4,744 (19.9)	4,595 (19.3)	4,351 (18.3)
1–2 time/week	23,887 (25.1)	5,751 (24.1)	6,028 (25.3)	5,997 (25.2)	6,111 (25.7)
3–4 time/week	24,809 (26.1)	5,469 (23.0)	6,153 (25.8)	6,465 (27.2)	6,722 (28.3)
>4 time/week	21,770 (22.9)	5,287 (22.2)	5,619 (23.6)	5,500 (23.1)	5,364 (22.6)
Pre-existing chronic conditions					
Cardiovascular disease	5,498 (5.8)	2,451 (10.3)	1,402 (5.9)	1,034 (4.3)	611 (2.6)
Hypertension	25,782 (27.1)	9,415 (39.5)	6,832 (28.7)	5,488 (23.1)	4,047 (17.0)
Diabetes	4,408 (4.6)	2,216 (9.3)	1,030 (4.3)	721 (3.0)	441 (1.9)
Regular use of NSAIDs^b^	23,785 (25.0)	6,987 (29.3)	6,035 (25.3)	5,614 (23.6)	5,149 (21.7)
Antibiotic use in past 3 months	1,289 (1.4)	310 (1.3)	313 (1.3)	335 (1.4)	331 (1.4)
Female-specific variables					
Postmenopause	36,279 (67.6)	8,552 (71.9)	9,446 (69.7)	9,422 (67.1)	8,859 (62.7)
Parity					
0	11,504 (21.4)	2,509 (21.1)	2,850 (21.0)	2,988 (21.3)	3,157 (22.3)
1–2	30,601 (57.1)	6,765 (56.9)	7,765 (57.3)	8,033 (57.2)	8,038 (56.9)
3+	11,527 (21.5)	2,622 (22.0)	2,935 (21.7)	3,028 (21.6)	2,942 (20.8)
Ever use of oral contraceptives	45,778 (85.4)	9,767 (82.1)	11,553 (85.3)	12,131 (86.3)	12,327 (87.2)
Ever use of HRT	20,852 (38.9)	5,639 (47.4)	5,684 (41.9)	5,216 (37.1)	4,313 (30.5)
LPA (min/week)	2121.9 (690.2)	1526.9 (464.3)	2005.8 (478.1)	2296.8 (549.9)	2659.6 (687.0)
MVPA (min/week)	290.1 (243.3)	145.5 (127.5)	233.0 (167.7)	310.2(207.0)	471.9 (303.3)
Sedentary time (min/week)	3949.8 (763.8)	4447.8 (732.1)	4094.6 (633.9)	3834.5 (622.0)	3421.2 (668.1)

Continuous variables are presented as mean (SD); categorical variables are presented as n (%).

^a^Townsend deprivation index = a composite area-level measure of deprivation based on unemployment, non-car ownership, non-home ownership, and household overcrowding; a higher score indicates higher deprivation.

^b^Regular NSAID use was assessed through a questionnaire. Participants were asked about their regular use (defined as most days of the week for the last 4 weeks) of common over-the-counter medications, including various brand names for aspirin, ibuprofen, paracetamol, and codeine. The questionnaire provided a comprehensive list of brand names to ensure accurate reporting of NSAID use, including combinations and less commonly recognized trade names.

BMI, Body Mass Index; NSAID, nonsteroidal anti-inflammatory drug; HRT, hormone replacement therapy; LPA, light physical activity; MVPA, moderate-to-vigorous physical activity.

Over 8.86 years median follow-up (interquartile range, 8.30–9.37), 247 incident UC cases were recorded. Participants were stratified into quartiles (Q1–Q4) based on accelerometer-measured Total mean PA volume levels (Table 1). Compared to the least active quartile (Q1), the most active quartile (Q4) comprised younger individuals with lower BMI and a higher proportion of females. An inverse trend in chronic disease (including cardiovascular disease, hypertension, and diabetes) prevalence was observed from Q1 to Q4. Q4 reported less NSAIDs and hormone replacement therapy use but higher oral contraceptive use. While differences in education level, material deprivation, smoking status, and alcohol consumption were noted across quartiles, these socioeconomic and lifestyle factors showed less pronounced trends compared to the health indicators.

### 3.2 Association of PA intensity and sedentary time with incident UC

Restricted cubic spline Cox regression revealed a U-shaped association between TPA and UC risk (*P* for non-linearity = 0.0018, Figure 2), with the nadir at approximately 30.9 milligravity (aHR: 0.54). LPA showed no significant association with UC risk, while MVPA demonstrated a significant dose–response relationship (*P* for trend = 0.004). Compared to the lowest MVPA quartile, the third (231.5 to <402.3 min/week) and highest quartiles (> 402.3 min/week) had aHRs of 0.53 and 0.57 (95% CI: 0.35–0.82 and 0.37–0.87). The magnitude of risk reduction was not substantially different between these two highest quartiles, suggest that a potential plateauing of the protective effect at higher activity levels. Sedentary time showed no significant link to UC risk (all in Table 2). These associations remained materially unchanged in mutually adjusted models simultaneously accounting for LPA, MVPA, and sedentary time (Supplementary Table 2).

**Figure 2:**
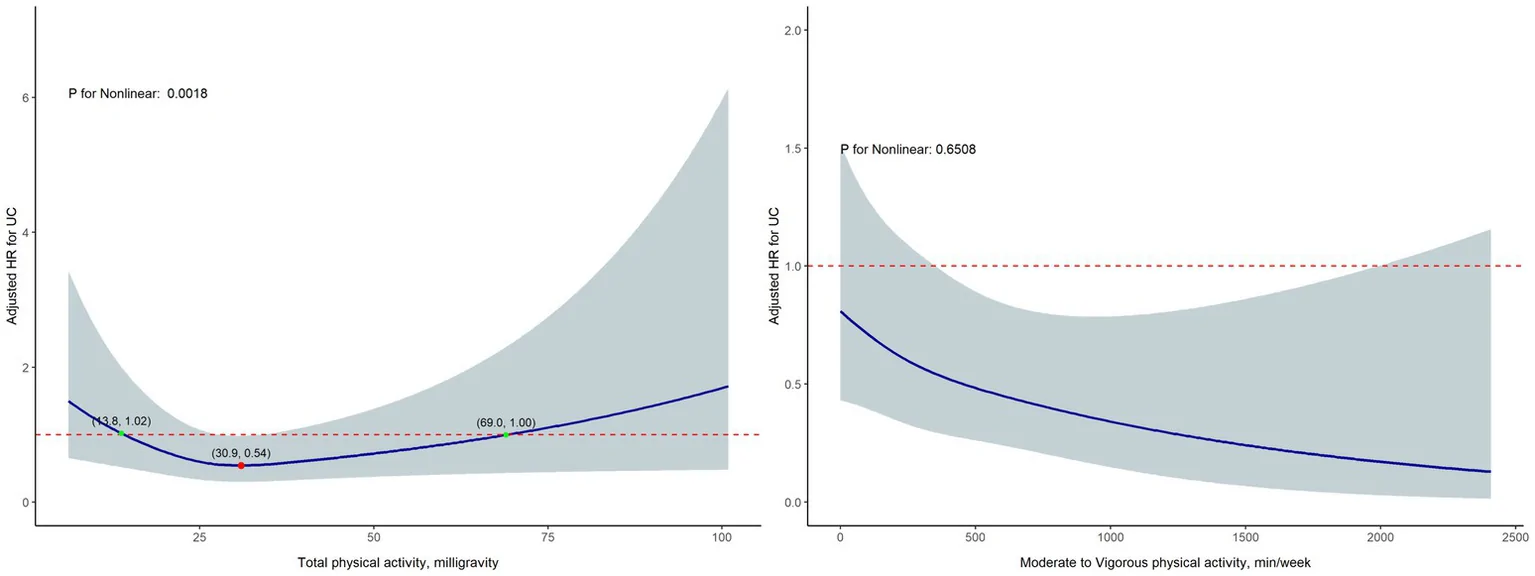
Restricted cubic spline for the association between accelerometer-measured average total volume, and moderate-to-vigorous-intensity physical activities and the risk of UC incidence. All adjusted for age, sex, body mass index, Townsend Deprivation Index, educational level, smoking status, alcohol consumption, and pre-existing chronic conditions (hypertension, diabetes and cardiovascular disease), non-steroid anti-inflammatory drug use, antibiotics use, and, among female participants, use of oral contraception, menopausal status, hormone therapy and parity. The solid line is the estimate (HR) and the shaded area is the 95% CI. HR indicates hazard ratio; CI, confidence interval; UC, ulcerative colitis.

**Table 2:** Association of light, moderate-to-vigorous physical activity and sedentary time with the risk of incident ulcerative colitis.

Exposure	Total	Events (rate^a^)	Crude Model	Adjusted Model^b^
			HR (95%CI), *p*-value	HR (95%CI), *P*-value
Light physical activity, min/week				
Q1 (<1634.9)	23,675	66 (3.2)	Ref	Ref
Q2 (1634.9- to <2068.7)	23,668	64 (3.1)	0.97 (0.66, 1.43), 0.873	1.06 (0.72, 1.58), 0.758
Q3 (2068.7- to <2552.1)	23,677	48 (2.3)	0.68 (0.44, 1.04), 0.0736	0.77 (0.49, 1.19), 0.241
Q4 (>2552.1)	23,656	65 (3.1)	0.94 (0.64, 1.40), 0.774	1.10 (0.73, 1.66), 0.658
*P* for trend			0.437	0.986
Moderate to vigorous physical activity, min/week				
Q1 (<112.5)	23,743	81 (4.0)	Ref	Ref
Q2 (112.5- to <231.5)	23,597	65 (3.2)	0.75 (0.52, 1.08), 0.125	0.78 (0.53, 1.13), 0.188
Q3 (231.5- to <402.3)	23,689	46 (2.2)	0.52 (0.34, 0.78), 0.00174	0.53 (0.35, 0.82), 0.00426
Q4 (>402.3)	23,647	51 (2.5)	0.56 (0.37, 0.84), 0.00486	0.57 (0.37, 0.87), 0.00991
*P* for trend			0.0009	0.00262
Sedentary time, min/week				
Q1 (<3437.9)	23,673	58 (2.8)	Ref	Ref
Q2 (3437.9- to <3950.9)	23,672	53 (2.6)	1.00 (0.65, 1.54), 0.992	0.99 (0.64, 1.52), 0.964
Q3 (3950.9- to <4457.8)	23,668	66 (3.2)	1.17 (0.78, 1.77), 0.448	1.13 (0.75, 1.72), 0.555
Q4 (>4457.8)	23,663	66 (3.2)	1.26 (0.84, 1.89), 0.269	1.11 (0.73, 1.70), 0.621
*P* for trend			0.196	0.508

^a^Incidence rates per 10,000 person-years.

^b^Adjusted for age, sex, body mass index, Townsend Deprivation Index, educational level, smoking status, alcohol consumption, and pre-existing chronic conditions (hypertension, diabetes and cardiovascular disease), non-steroid anti-inflammatory drug use, antibiotics use, and, among female participants, use of oral contraception, menopausal status, hormone therapy and parity.

HR, hazard ratio; CI, confidence interval; Ref, reference.

These findings remained consistent across a 2-year blanking period (Supplementary Table 3) and in competing risk analyses considering death (Supplementary Table 4). The null association for sedentary time was also confirmed in the mutually adjusted model (Supplementary Table 2). The PA-UC relationship was not significantly modified by sex, age (<65, ≥65), BMI (<25, ≥25), TDI (<median, ≥median), smoking status (never, current or former), or CVD status (yes, no) (all *P* for interaction >0.1) (Supplementary Tables 5–10).

### 3.3 Association of MVPA pattern with incident UC

After identifying a significant link between MVPA and UC risk, we investigated optimal MVPA patterns. The UK National Health Service advises distributing MVPA throughout the week (18). However, the benefits of concentrating exercise into 1–2 days [“weekend warrior” pattern (21)] compared to more consistent activity remain unclear. We analyzed 94,099 participants with complete daily MVPA data, excluding 1,089 individuals from the initial cohort of 95,197 due to incomplete records (Supplementary Tables 11, 12). Based on a median MVPA threshold of ≥231.5 min/week, we classified 26,235 (27.9%) participants as active weekend warriors, 20,832 (22.1%) as active regular, and 47,032 (50.0%) as inactive (Supplementary Table 12). Active weekend warriors demonstrated substantially higher MVPA on their two most active days versus the other five (Figure 3 and Supplementary Figure 1). In contrast, the active regular group exhibited more consistent MVPA distribution throughout the week.

**Figure 3:**
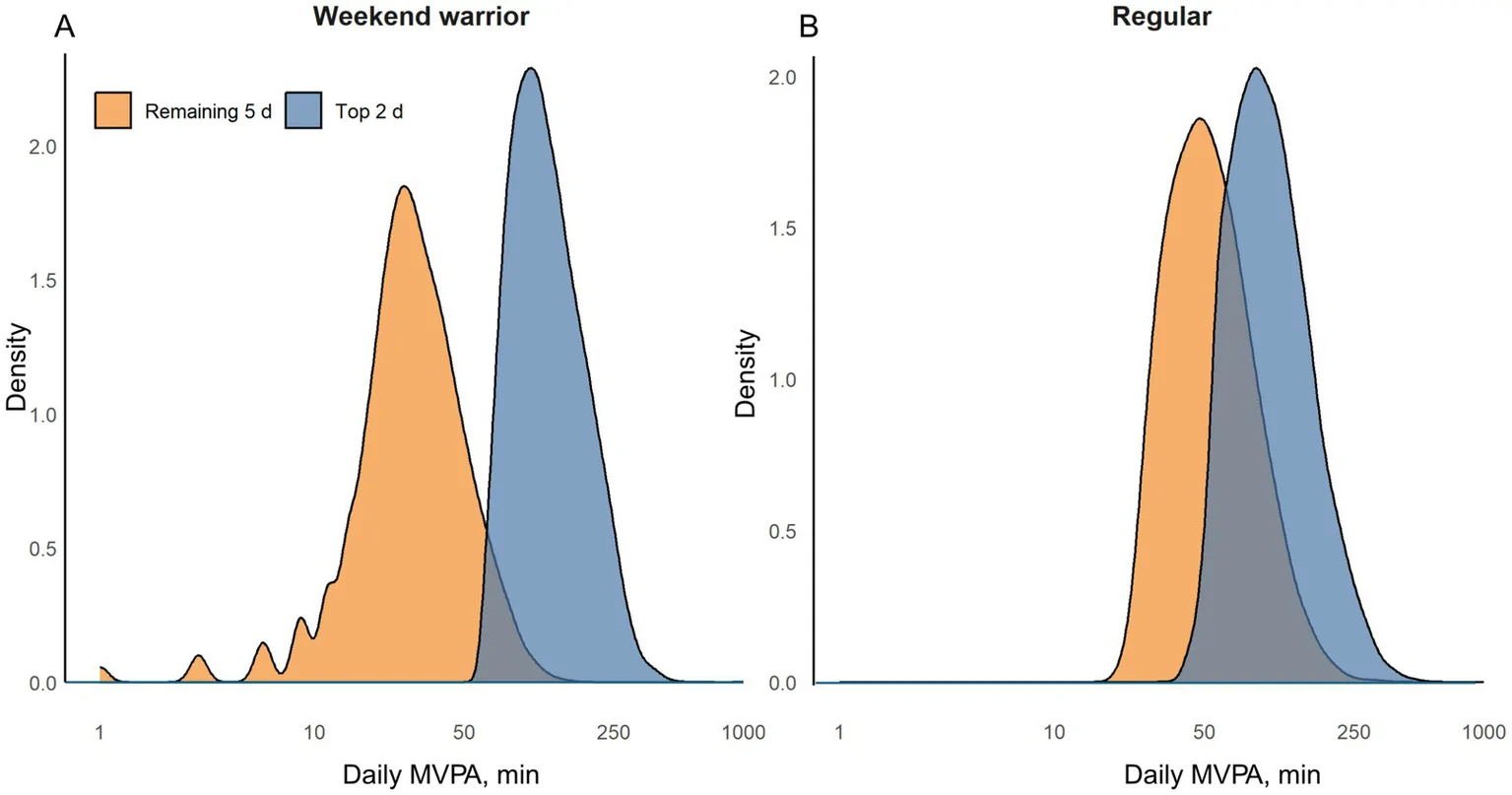
Weekly MVPA distribution: top 2 days vs. remaining 5 days in active individuals (active: ≥231.5 min/week MVPA, sample median). MVPA distribution for active individuals (≥231.5 min/week, *n* = 47,067): 2 most active days (blue) vs. remaining 5 (yellow). **(A)** Weekend warriors (≥50% MVPA in 1–2 days). **(B)** Regular actives. Note: 147 weekend warriors (0.6%) with zero MVPA on 5 days assigned 1 min for log scale. MVPA indicates moderate to vigorous physical activity.

Multivariable-adjusted models revealed that compared to inactive individuals, both weekend warrior and regular activity patterns correlated with reduced risk of UC (Table 3). This association remained consistent across various MVPA thresholds (Table 3). At the 25th percentile threshold (≥112.5 min/week), both weekend warriors (aHR 0.74, 95% CI 0.55–0.99) and regular activity participants (aHR 0.59, 95% CI 0.41–0.84) showed significantly reduced UC risk. Similarly, at the 50th percentile (≥231.5 min/week), weekend warriors (aHR 0.72, 95% CI 0.53–0.99) and regular activity (aHR 0.57, 95% CI 0.39–0.82) were protective. For the guideline-based threshold (≥150 min/week), only regular activity was significantly protective (aHR 0.63, 95% CI 0.44–0.90). At the highest threshold (≥402.0 min/week), trends persisted but lost statistical significance. Event rates were comparable between the active weekend warrior and active regular groups (Figures 4A,B). When stratified by MVPA deciles, the weekend warrior pattern did not significantly differ from the regular activity pattern in terms of UC risk (aHR 1.08, 95% CI 0.78–1.48). Analyses using a 2-year blanking period and competing risk models (accounting for death) further supported the main findings (Supplementary Tables 13, 14). Notably, analyses with the 2-year blanking period demonstrated more pronounced risk reduction at lower thresholds (Supplementary Table 13). Overall, regular activity consistently demonstrated the lowest aHRs across all thresholds, suggesting more consistent protection against UC risk.

**Table 3:** Associations between physical activity pattern and incident ulcerative colitis across varying MVPA threshold^a^.

Activity threshold	Activity Pattern^b^	Event (rate^c^)	Hazard ratio (95% CI)	*p*_value
25th percentile (≥112.5 min MVPA/week)	Inactive (*n* = 23,480)	81 (4.01)	Ref	Ref
	Weekend warrior (*n* = 45,559)	111 (2.78)	0.74 (0.55–0.99)	0.046
	Regular activity (*n* = 25,060)	51 (2.32)	0.59 (0.41–0.84)	0.004
Guideline-based (≥150 min MVPA/week)	Inactive (*n* = 31,414)	101 (3.73)	Ref	Ref
	Weekend warrior (*n* = 38,772)	93 (2.74)	0.78 (0.58–1.04)	0.091
	Regular activity (*n* = 23,913)	49 (2.34)	0.63 (0.44–0.90)	0.011
50th percentile (≥231.5 min MVPA/week)	Inactive (*n* = 47,032)	147 (3.61)	Ref	Ref
	Weekend warrior (*n* = 26,235)	58 (2.52)	0.72 (0.53–0.99)	0.043
	Regular activity (*n* = 20,832)	38 (2.08)	0.57 (0.39–0.82)	0.003
75th percentile (≥402.0 min MVPA/week)	Inactive (*n* = 70,568)	194 (3.16)	Ref	Ref
	Weekend warrior (*n* = 11,044)	25 (2.58)	0.85 (0.55–1.30)	0.449
	Regular activity (*n* = 12,487)	24 (2.18)	0.69 (0.45–1.06)	0.088

^a^Adjusted for age, sex, body mass index, townsend deprivation index, educational level, smoking status, alcohol consumption, and pre-existing chronic conditions (hypertension, diabetes and cardiovascular disease), non-steroid anti-inflammatory drug use, antibiotics use, and, among female participants, use of oral contraception, menopausal status, hormone therapy and parity.

^b^Inactive defined as weekly MVPA below the specified threshold.

^c^Incidence rates per 10,000 person-years.

MVPA, moderate-to-vigorous physical activity; CI, confidence interval; Ref, reference.

**Figure 4:**
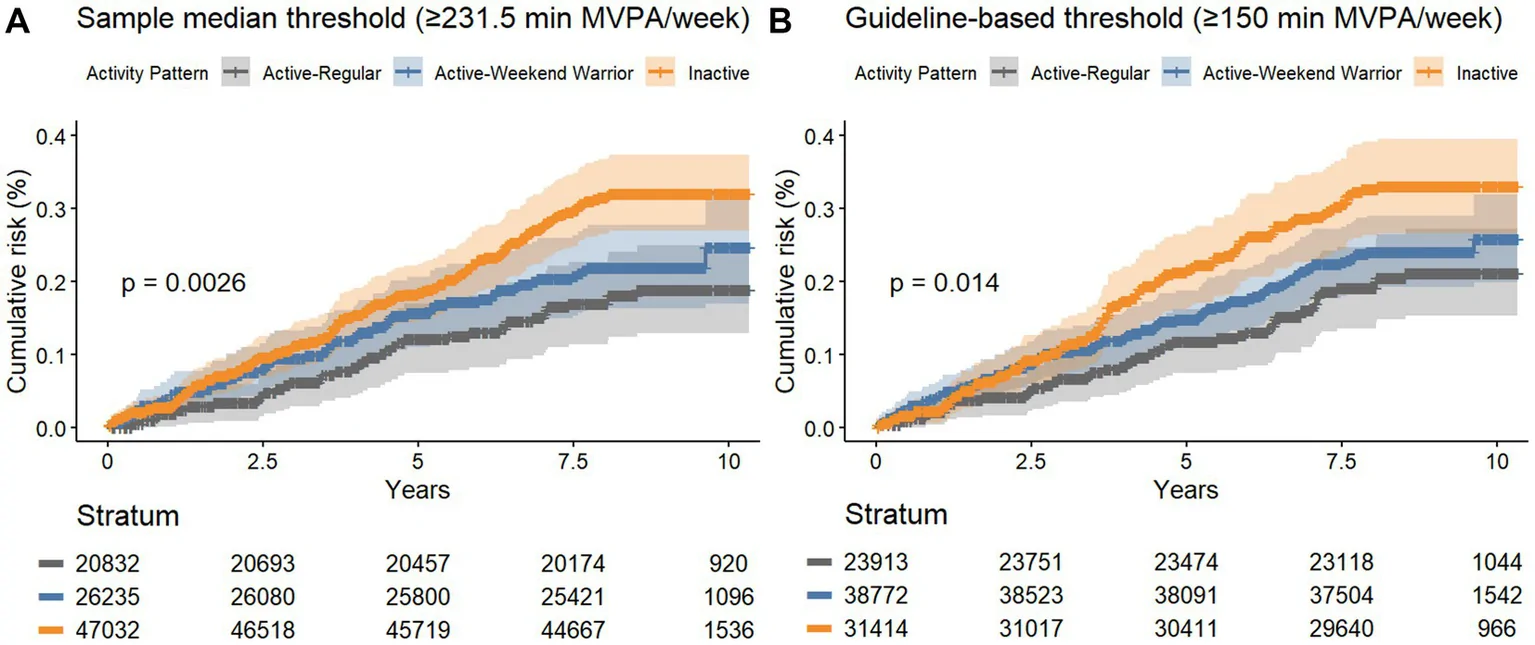
Cumulative risk of incident UC events stratified by activity pattern. Crude cumulative risk of incident UC events across accelerometer-derived activity patterns: Inactive (orange), Active-Weekend Warrior (blue), and Active-Regular (gray). Panel **(A)** uses the sample median activity threshold (≥231.5 min MVPA/week), while Panel **(B)** uses the guideline-based threshold (≥150 min MVPA/week). At-risk numbers are shown below each plot. Shaded areas represent 95% confidence intervals. UC, ulcerative colitis; MVPA, moderate-to-vigorous physical activity.

## 4 Discussion

### 4.1 Main findings

This large-scale cohort study found MVPA significantly lowered UC risk after adjusting for potential confounders. A dose–response relationship was observed, with 231.5 to over 402.3 min of weekly MVPA (such as swimming, jogging, stationary cycling, or other aerobic activities) associated with a 37–41% lower adjusted UC risk compared to the lowest quartile. Notably, this protective effect plateaued at higher activity levels, suggesting that moderate MVPA may suffice for optimal UC risk reduction. Adjusted analyses showed no significant associations between UC risk and either LPA or sedentary time. The protective effect of MVPA remained significant after mutual adjustment for LPA and sedentary time, confirming its independent role in UC risk reduction. Both consistent weekly MVPA and the “weekend warrior” approach (concentrating MVPA into 1–2 days per week) were associated with reduced UC risk in multivariable-adjusted models. These findings remained robust across various sensitivity analyses and subgroup examinations.

### 4.2 Clinical implications

These results offer significant implications for UC prevention strategies. Firstly, the study revealed an inverse dose–response link between MVPA and UC incidence risk, with 231.5–402.3 min of MVPA per week significantly reducing UC incidence risk by 41% (aHR: 0.59, 95% CI: 0.40–0.85). It is suggested that adults engage in 231.5–402.3 min of MVPA weekly. These accelerometer-based measurements provide valuable reference for developing UC prevention guidelines. Notably, a potential plateau effect in protective benefits was observed at higher activity levels (>402.3 min/week), suggesting there may be an optimal range of activity (Table 2). Secondly, this study is the first to compare the impact of the “weekend warrior” pattern and regular exercise patterns on UC risk. Results indicated that both patterns were associated with reduced UC risk across various MVPA thresholds. At the 231.5 min per week threshold, both weekend warriors and regular activity demonstrated significant protective effects. Direct comparison between the two active patterns showed no significant difference (aHR 1.08, 95% CI: 0.78–1.48), suggesting that the protective effects are broadly comparable despite the slightly lower point estimate for regular activity. For those with hectic weekdays, weekend-concentrated exercise may still offer health benefits. However, the regular activity pattern consistently showed the lowest aHRs across all thresholds, indicating it may be the most ideal exercise pattern, aligning with existing public health recommendations (5). However, given the observational nature of our study and the relatively small number of UC events in subgroup analyses, this finding should be interpreted with caution and warrants confirmation in future prospective studies with larger case numbers. Overall, this study strongly supports MVPA as a potential UC prevention strategy, with both weekend warrior and regular exercise regimens showing protective effects.

### 4.3 Comparison with other studies and implications

Our findings align with a recent meta-analysis (3 cohort, 7 case–control studies) showing high PA significantly lowered UC risk versus low activity (relative risk [RR]: 0.62 [95% CI: 0.43, 0.88], *p* = 0.008) (22). Similarly, a study objectively measuring physical fitness in over 240,000 Swedish adolescent males found an inverse correlation between physical fitness and UC risk (23). However, these findings diverge from several questionnaire-based prospective cohort studies that found no association between PA and UC risk (8, 10, 11). Indeed, the variability in PA definitions across different studies makes comparisons challenging (24). Questionnaire-based surveys not only suffer from recall bias but also have limited reliability (12). For instance, the frequent, yet irregular occurrence of LPA challenges its precise measurement through self-reported surveys.

The protective effect of MVPA (such as swimming, jogging, stationary cycling, or other aerobic activities) on UC risk likely results from multiple interacting factors. Animal studies have shown that MVPA reduces chronic bowel inflammation by inhibiting local proinflammatory responses in DSS-induced colitis (25, 26). Additionally, MVPA may modulate the gut microbiome, which is pivotal in regulating exercise-induced oxidative stress, inflammation, and metabolism (27). Notably, the conversion of exercise-induced lactate to propionate by specific microbes may be protective (28), given that lower propionate levels have been observed in UC patients (29). Furthermore, β-hydroxybutyrate, produced during aerobic exercise, contributes to this effect by promoting regulatory T cell differentiation, thus reducing the Th17/Treg ratio (30). PA may also activate autophagy, a process known to prevent various inflammatory conditions (31). These multifaceted pathways likely work synergistically to confer protection against UC development.

### 4.4 Limitations

This study has several limitations. Firstly, the UK Biobank cohort’s older, predominantly European demographic may bias findings toward late-onset IBD and limit generalizability. Secondly, the optimal MVPA threshold for wrist-worn accelerometers remains uncertain. Thirdly, as an observational study, we cannot definitively rule out residual confounding or reverse causality, despite adjusting for numerous confounders. Notably, our analytical model did not incorporate all potential dietary factors (e.g., fiber, red or processed meat intake), which may have contributed to residual confounding. Additionally, temporal gap between accelerometer data and covariates may affect baseline accuracy, despite using most temporally proximate covariate data and known variable stability (13). Fifth, the one-week measurement period may not fully represent long-term activity patterns, although studies have shown moderate to high correlations in repeated assessments over extended periods (32–34). Sixth, our MVPA classification method, while validated (16), may underestimate non-ambulatory activities. Lastly, we did not account for the potential impact of daily timing of PA on health outcomes, an important consideration for future research.

## 5 Conclusion

In this large prospective cohort of approximately 95,000 UK Biobank participants, accelerometer-measured MVPA was associated with a significantly reduced risk of incident UC over a median follow-up of 8.86 years. The protective association followed a non-linear dose–response pattern, with risk reduction appearing to plateau at higher activity levels. Notably, both the ‘weekend warrior’ pattern (concentrated activity within 1–2 days) and regular daily MVPA were associated with comparable risk reductions, suggesting that accumulated MVPA—rather than its weekly distribution—may be the key determinant of UC prevention. While MVPA represents a promising modifiable target for UC primary prevention, further studies are warranted to validate optimal thresholds in diverse populations.

## Data Availability

Publicly available datasets were analyzed in this study. The data that support the findings of this study are available from UK Biobank (https://www.ukbiobank.ac.uk/enable-your-research/register) but restrictions apply to availability. Data access requires application and approval through UK Biobank’s Access Management System.
