# Supplementary material for: Accelerometer-measured physical activity and risk of incident ulcerative colitis: a population-based prospective study
Source: Front Public Health. 2026 Sep 22;14:1955306. doi: 10.3389/fpubh.2026.1955306 (PMC13638607; doi:10.3389/fpubh.2026.1955306)
Supplement: Supplementary file 1 [file Data_Sheet_1.docx]

Supplementary Table 1. The percentages of participants with missing covariates involved in accelerometer-based physical activity analysis.

| Characteristic | Number | Percentage of missing data |
| --- | --- | --- |
| Age | 0 | 0.00% |
| Sex | 0 | 0.00% |
| Body mass index (BMI) | 156 | 0.16% |
| Townsend Index score^a^ | 110 | 0.12% |
| Education (college or university) | 0 | 0.00% |
| Cigarette smoking | 232 | 0.24% |
| Alcohol consumption | 62 | 0.07% |
| Hypertension history | 0 | 0.00% |
| Diabetes history | 0 | 0.00% |
| Cardiovascular disease history | 0 | 0.00% |
| Menopause | 0 | 0.00% |
| parity | 33 | 0.06% |
| Ever use of oral contraceptives | 0 | 0.00% |
| Ever use of hormone replacement therapy | 0 | 0.00% |
| Regular use of NSAID (%) | 0 | 0.00% |
| Antibiotics use for last 3 months | 0 | 0.00% |
| Light intensity | 0 | 0.00% |
| Moderate to Vigorous intensity | 0 | 0.00% |
| Sedentary time | 0 | 0.00% |
| Mean accelerometer activity | 0 | 0.00% |

^a^ Townsend deprivation index =a composite area-level measure of deprivation based on unemployment, non-car ownership, non-home ownership, and household overcrowding; a higher score indicates higher deprivation.

Supplementary Table 2. Mutually adjusted associations of LPA, MVPA, and sedentary time with incident UC risk^a^

| Exposure | Q2 HR (95% CI) | P | Q3 HR (95% CI) | P | Q4 HR (95% CI) | P | P for trend |
| --- | --- | --- | --- | --- | --- | --- | --- |
| LPA | 1.05 (0.73-1.51) | 0.779 | 0.88 (0.58-1.34) | 0.555 | 1.15 (0.73-1.82) | 0.544 | 0.811 |
| MVPA | 0.82 (0.59-1.15) | 0.254 | 0.59 (0.41-0.86) | 0.006 | 0.64 (0.43-0.94) | 0.023 | 0.005 |
| Sedentary time | 0.92 (0.62-1.36) | 0.682 | 1.12 (0.74-1.71) | 0.581 | 1.03 (0.64-1.66) | 0.9 | 0.848 |

^a^ Model adjusted for age, sex, BMI, Townsend Deprivation Index, educational level, smoking status, alcohol consumption, pre-existing chronic conditions (hypertension, diabetes and cardiovascular disease), NSAID use, antibiotics use, and female-specific variables (menopausal status, parity, contraceptive use, and hormone replacement therapy). LPA, MVPA, and sedentary time were mutually adjusted in the same model.

HR, hazard ratio; CI, confidence interval; LPA, light physical activity; MVPA, moderate-to-vigorous physical activity; UC, ulcerative colitis; Ref, reference. Physical activity and sedentary time are presented in minutes per week (min/week). Q2, Q3, and Q4 indicate the second, third, and fourth quartiles, respectively, with Q1 as the reference group. The model was adjusted for the same covariates as in Table 2, with LPA, MVPA, and sedentary time mutually included in a single Cox model to estimate their independent associations.

Supplemental Table 3. Sensitivity analysis on the association of physical activity and sedentary time with incident ulcerative colitis by excluding events that occurred within the first two years of follow-up

| Types of physical activity | Total | Events (rate^a^) | Crude Model | Adjusted Model^b^ |
| --- | --- | --- | --- | --- |
|  |  |  | HR (95%CI), *P* value | HR (95%CI), *P* value |
| Light physical activity, min/week | | | |  |
| Q1 (<1634.9) | 23675 | 66 (3.2) | ref | ref |
| Q2 (1634.9-to<2068.7) | 23668 | 64 (3.1) | 0.97 (0.66, 1.43), 0.873 | 1.06 (0.72, 1.58), 0.758 |
| Q3 (2068.7-to<2552.1) | 23677 | 48 (2.3) | 0.68 (0.44, 1.04), 0.0736 | 0.77 (0.49, 1.19), 0.241 |
| Q4 (>2552.1) | 23656 | 65 (3.1) | 0.94 (0.64, 1.40), 0.774 | 1.10 (0.73, 1.66), 0.658 |
| *P* for trend |  |  | 0.437 | 0.986 |
| Moderate to vigorous physical activity, min/week | | | |  |
| Q1 (<112.5) | 23743 | 81 (4) | ref | ref |
| Q2 (112.5-to<231.5) | 23597 | 65 (3.2) | 0.75 (0.52, 1.08), 0.125 | 0.78 (0.53, 1.13), 0.188 |
| Q3 (231.5-to<402.3) | 23689 | 46 (2.2) | 0.52 (0.34, 0.78), 0.00174 | 0.53 (0.35, 0.82), 0.00426 |
| Q4 (>402.3) | 23647 | 51 (2.5) | 0.56 (0.37, 0.84), 0.00486 | 0.57 (0.37, 0.87), 0.00991 |
| *P* for trend |  |  | <0.001 | 0.003 |
| Sedentary time, min/week | | | |  |
| Q1 (<3437.9) | 23673 | 58 (2.8) | ref | ref |
| Q2 (3437.9-to<3950.9) | 23672 | 53 (2.6) | 1.00 (0.65, 1.54), 0.992 | 0.99 (0.64, 1.52), 0.964 |
| Q3 (3950.9-to<4457.8) | 23668 | 66 (3.2) | 1.17 (0.78, 1.77), 0.448 | 1.13 (0.75, 1.72), 0.555 |
| Q4 (>4457.8) | 23663 | 66 (3.2) | 1.26 (0.84, 1.89), 0.269 | 1.11 (0.73, 1.70), 0.621 |
| *P* for trend |  |  | 0.196 | 0.508 |

^a^ Incidence rates per 10000 person-years.

^b^ Adjusted for age, sex, body mass index, Townsend Deprivation Index, educational level, smoking status, alcohol consumption, and pre-existing chronic conditions (hypertension, diabetes and cardiovascular disease), non-steroid anti-inflammatory drug use, antibiotics use, and, among female participants, use of oral contraception, menopausal status, hormone therapy and parity.

HR, hazard ratio; CI, confidence interval; Ref, reference.

Supplemental Table 4. Sensitivity analysis on the association of physical activity and sedentary time with incident ulcerative colitis based on competitive risk model (Fine and Gray)

| Types of physical activity | Total | Events (rate^a^) | No. of Death | Crude Model | Adjusted Model^b^ |
| --- | --- | --- | --- | --- | --- |
|  |  |  |  | SHR (95%CI), *P* value | SHR (95%CI), *P* value |
| Light physical activity, min/week | | |  |  |  |
| Q1 (<1634.9) | 23675 | 66 (3.2) | 1464 | ref | ref |
| Q2 (1634.9-to<2068.7) | 23668 | 64 (3.1) | 968 | 0.97 (0.69, 1.37), 0.86 | 1.04 (0.74, 1.47), 0.8 |
| Q3 (2068.7-to<2552.1) | 23677 | 48 (2.3) | 819 | 0.73 (0.50, 1.05), 0.093 | 0.80 (0.55, 1.17), 0.25 |
| Q4 (>2552.1) | 23656 | 65 (3.1) | 728 | 0.99 (0.70, 1.39), 0.94 | 1.10 (0.77, 1.56), 0.6 |
| *P* for trend |  |  |  | 0.6 | 0.94 |
| Moderate to vigorous physical activity, min/week | | | |  |  |
| Q1 (<112.5) | 23743 | 81 (4) | 1509 | ref | ref |
| Q2 (112.5-to<231.5) | 23597 | 65 (3.2) | 933 | 0.81 (0.58, 1.12), 0.2 | 0.83 (0.60, 1.15), 0.27 |
| Q3 (231.5-to<402.3) | 23689 | 46 (2.2) | 846 | 0.57 (0.40, 0.82), 0.0022 | 0.59 (0.40, 0.85), 0.005 |
| Q4 (>402.3) | 23647 | 51 (2.5) | 691 | 0.63 (0.44, 0.90), 0.01 | 0.63 (0.44, 0.92), 0.017 |
| *P* for trend |  |  |  | 0.0025 | 0.0054 |
| Sedentary time, min/week | |  |  |  |  |
| Q1 (<3437.9) | 23673 | 58 (2.8) | 749 | ref | ref |
| Q2 (3437.9-to<3950.9) | 23672 | 53 (2.6) | 856 | 0.91 (0.63, 1.33), 0.64 | 0.92 (0.63, 1.33), 0.65 |
| Q3 (3950.9-to<4457.8) | 23668 | 66 (3.2) | 965 | 1.14 (0.80, 1.62), 0.47 | 1.13 (0.80, 1.60), 0.49 |
| Q4 (>4457.8) | 23663 | 66 (3.2) | 1409 | 1.14 (0.80, 1.62), 0.47 | 1.06 (0.75, 1.51), 0.74 |
| *P* for trend |  |  |  | 0.29 | 0.5 |

^a^ Incidence rates per 10000 person-years.

^b^ Adjusted for age, sex, body mass index, Townsend Deprivation Index, educational level, smoking status, alcohol consumption, and pre-existing chronic conditions (hypertension, diabetes and cardiovascular disease), non-steroid anti-inflammatory drug use, antibiotics use, and, among female participants, use of oral contraception, menopausal status, hormone therapy and parity.

SHR, subdistribution hazard ratio; CI, confidence interval; Ref, reference.

Supplemental Table 5. Risk of incident ulcerative colitis according to physical activity and sedentary time stratified by sex categories^a^

| Subgroup | Men | | | Women | | | *P* for interaction^c^ |
| --- | --- | --- | --- | --- | --- | --- | --- |
| Types of physical activity | Total | Events (rate^b^) | aHR (95%CI) | Total | Events (rate^b^) | aHR (95%CI) |  |
| Light physical activity, min/week | | |  |  |  |  | 0.802 |
| Q1 (<1634.9) | 14731 | 45 (3.6) | Ref | 8944 | 21 (2.7) | Ref |  |
| Q2 (1634.9-to<2068.7) | 11069 | 33 (3.4) | 1.01 (0.64, 1.58) | 12599 | 31 (2.8) | 1.07 (0.61, 1.86) | |
| Q3 (2068.7-to<2552.1) | 8652 | 18 (2.4) | 0.70 (0.40, 1.21) | 15025 | 30 (2.3) | 0.86 (0.49, 1.51) | |
| Q4 (>2552.1) | 6884 | 26 (4.3) | 1.21 (0.74, 1.98) | 16772 | 39 (2.6) | 1.01 (0.59, 1.74) | |
| Moderate to vigorous physical activity, min/week | | | |  |  |  | 0.175 |
| Q1 (<112.5) | 7629 | 29 (4.5) | Ref | 16114 | 52 (3.7) | Ref |  |
| Q2 (112.5-to<231.5) | 9105 | 38 (4.8) | 1.16 (0.71, 1.90) | 14492 | 27 (2.1) | 0.59 (0.37, 0.95) | |
| Q3 (231.5-to<402.3) | 10776 | 24 (2.6) | 0.64 (0.36, 1.11) | 12913 | 22 (1.9) | 0.55 (0.33, 0.93) | |
| Q4 (>402.3) | 13826 | 31 (2.6) | 0.65 (0.38, 1.11) | 9821 | 20 (2.3) | 0.66 (0.39, 1.14) | |
| Sedentary time, min/week | |  |  |  |  |  | 0.258 |
| Q1 (<3437.9) | 7993 | 18 (2.6) | Ref | 15680 | 40 (2.9) | Ref |  |
| Q2 (3437.9-to<3950.9) | 9076 | 26 (3.3) | 1.33 (0.73, 2.42) | 14596 | 27 (2.1) | 0.72 (0.44, 1.18) | |
| Q3 (3950.9-to<4457.8) | 10721 | 38 (4.1) | 1.66 (0.94, 2.92) | 12947 | 28 (2.5) | 0.85 (0.52, 1.39) | |
| Q4 (>4457.8) | 13546 | 40 (3.4) | 1.33 (0.76, 2.34) | 10117 | 26 (3) | 0.99 (0.60, 1.66) | |

^a^ Adjusted for age, body mass index, Townsend Deprivation Index, educational level, smoking status, alcohol consumption, and pre-existing chronic conditions (hypertension, diabetes and cardiovascular disease), non-steroid anti-inflammatory drug use, antibiotics use, and, among female participants, use of oral contraception, menopausal status, hormone therapy and parity.

^b^ Incidence rates per 10000 person-years.

^c^ *P* for interaction, The interaction between sex and PA categories were exaimed by liklihood ratio test. Specifically, we established two models, one without the inclusion of interaction terms [quartiles of PA (factor), sex, and all covariates] and the other with the inclusion of interaction terms [quartiles of PA (factor), sex, quartiles of PA (factor) × sex, and all covariates]. Likelihood ratio tests were employed to compare the disparities between these two models, if the *P-*value is significant, it suggests a difference between the two models, indicating the potential presence of interaction effects.

aHR, Adjuster hazard ratio; CI, confidence interval; Ref, reference.

Supplemental Table 6. Risk of incident ulcerative colitis according to physical activity and sedentary time stratified by age categories^a^

| Subgroup | Age < 65 | | | Age ≥ 65 | | | *P* for interaction^c^ |
| --- | --- | --- | --- | --- | --- | --- | --- |
| Types of physical activity | Total | Events (rate^b^) | aHR (95%CI) | Total | Events (rate^b^) | aHR (95%CI) |  |
| Light physical activity, min/week | | | |  |  |  | 0.507 |
| Q1 (<1634.9) | 12926 | 29 (2.5) | Ref | 10749 | 37 (4.1) | Ref |  |
| Q2 (1634.9-to<2068.7) | 12712 | 30 (2.7) | 1.11 (0.66, 1.86) | 10956 | 34 (3.6) | 0.97 (0.61, 1.56) |  |
| Q3 (2068.7-to<2552.1) | 12590 | 27 (2.4) | 1.03 (0.60, 1.75) | 11087 | 21 (2.2) | 0.62 (0.36, 1.07) |  |
| Q4 (>2552.1) | 13502 | 34 (2.8) | 1.19 (0.71, 2.00) | 10154 | 31 (3.5) | 1.03 (0.62, 1.72) |  |
| Moderate to vigorous physical activity, min/week | | | |  |  |  | 0.083 |
| Q1 (<112.5) | 11570 | 36 (3.5) | Ref | 12173 | 45 (4.4) | Ref |  |
| Q2 (112.5-to<231.5) | 12534 | 34 (3.1) | 0.84 (0.52, 1.35) | 11063 | 31 (3.3) | 0.82 (0.51, 1.31) |  |
| Q3 (231.5-to<402.3) | 13578 | 17 (1.4) | 0.37 (0.20, 0.67) | 10111 | 29 (3.3) | 0.87 (0.53, 1.41) |  |
| Q4 (>402.3) | 14048 | 33 (2.6) | 0.64 (0.39, 1.06) | 9599 | 18 (2.2) | 0.58 (0.32, 1.03) |  |
| Sedentary time, min/week | | |  |  |  |  | 0.41 |
| Q1 (<3437.9) | 13185 | 30 (2.6) | Ref | 10488 | 28 (3.1) | Ref |  |
| Q2 (3437.9-to<3950.9) | 12359 | 23 (2.1) | 0.83 (0.48, 1.44) | 11313 | 30 (3.1) | 0.96 (0.58, 1.62) |  |
| Q3 (3950.9-to<4457.8) | 12841 | 32 (2.8) | 1.11 (0.67, 1.84) | 10827 | 34 (3.7) | 1.10 (0.66, 1.83) |  |
| Q4 (>4457.8) | 13345 | 35 (3.0) | 1.15 (0.69, 1.90) | 10318 | 31 (3.6) | 0.96 (0.56, 1.64) |  |

^a^ Adjusted for sex, body mass index, Townsend Deprivation Index, educational level, smoking status, alcohol consumption, and pre-existing chronic conditions (hypertension, diabetes and cardiovascular disease), non-steroid anti-inflammatory drug use, antibiotics use, and, among female participants, use of oral contraception, menopausal status, hormone therapy and parity.

^b^ Incidence rates per 10000 person-years.

^c^ *P* for interaction, The interaction between age and PA categories were exaimed by liklihood ratio test. Specifically, we established two models, one without the inclusion of interaction terms [quartiles of PA (factor), age, and all covariates] and the other with the inclusion of interaction terms [quartiles of PA (factor), age, quartiles of PA (factor) × age, and all covariates]. Likelihood ratio tests were employed to compare the disparities between these two models, if the *P-*value is significant, it suggests a difference between the two models, indicating the potential presence of interaction effects.

aHR, Adjuster hazard ratio; CI, confidence interval; Ref, reference.

Supplemental Table 7. Risk of incident ulcerative colitis according to physical activity and sedentary time stratified by BMI categories^a^

| Subgroup | BMI < 25 | | | BMI ≥ 25 | | | *P* for interaction^c^ |
| --- | --- | --- | --- | --- | --- | --- | --- |
| Types of physical activity | Total | Events (rate^b^) | aHR (95%CI) | Total | Events (rate^b^) | aHR (95%CI) |  |
| Light physical activity, min/week | | |  |  |  |  | 0.716 |
| Q1 (<1634.9) | 9322 | 22 (0.3) | Ref | 14349 | 46 (0.4) | Ref |  |
| Q2 (1634.9-to<2068.7) | 9319 | 23 (0.3) | 1.09 (0.60, 1.97) | 14349 | 41 (0.3) | 0.96 (0.63, 1.47) |  |
| Q3 (2068.7-to<2552.1) | 9320 | 26 (0.3) | 1.24 (0.69, 2.22) | 14350 | 25 (0.2) | 0.60 (0.37, 0.98) |  |
| Q4 (>2552.1) | 9321 | 19 (0.2) | 0.88 (0.47, 1.66) | 14346 | 41 (0.3) | 1.00 (0.65, 1.55) |  |
| Moderate to vigorous physical activity, min/week | | | |  |  |  | 0.168 |
| Q1 (<112.5) | 9345 | 31 (0.4) | Ref | 14351 | 47 (0.4) | Ref |  |
| Q2 (112.5-to<231.5) | 9299 | 23 (0.3) | 0.76 (0.44, 1.32) | 14346 | 39 (0.3) | 0.85 (0.55, 1.31) |  |
| Q3 (231.5-to<402.3) | 9326 | 22 (0.3) | 0.73 (0.42, 1.27) | 14359 | 35 (0.3) | 0.77 (0.49, 1.21) |  |
| Q4 (>402.3) | 9312 | 14 (0.2) | 0.45 (0.23, 0.86) | 14338 | 32 (0.3) | 0.68 (0.42, 1.09) |  |
| Sedentary time, min/week | |  |  |  |  |  | 0.391 |
| Q1 (<3437.9) | 9323 | 22 (0.3) | Ref | 14350 | 34 (0.3) | Ref |  |
| Q2 (3437.9-to<3950.9) | 9320 | 19 (0.2) | 0.87 (0.47, 1.60) | 14347 | 39 (0.3) | 1.16 (0.73, 1.84) |  |
| Q3 (3950.9-to<4457.8) | 9320 | 20 (0.2) | 0.93 (0.50, 1.70) | 14348 | 39 (0.3) | 1.14 (0.72, 1.81) |  |
| Q4 (>4457.8) | 9319 | 29 (0.4) | 1.31 (0.74, 2.32) | 14349 | 41 (0.3) | 1.12 (0.71, 1.78) |  |

^a^ Adjusted for age, sex, Townsend Deprivation Index, educational level, smoking status, alcohol consumption, and pre-existing chronic conditions (hypertension, diabetes and cardiovascular disease), non-steroid anti-inflammatory drug use, antibiotics use, and, among female participants, use of oral contraception, menopausal status, hormone therapy and parity.

^b^ Incidence rates per 10000 person-years.

^c^ *P* for interaction, The interaction between BMI and PA categories were exaimed by liklihood ratio test. Specifically, we established two models, one without the inclusion of interaction terms [quartiles of PA (factor), BMI, and all covariates] and the other with the inclusion of interaction terms [quartiles of PA (factor), BMI, quartiles of PA (factor) × BMI, and all covariates]. Likelihood ratio tests were employed to compare the disparities between these two models, if the *P-*value is significant, it suggests a difference between the two models, indicating the potential presence of interaction effects.

BMI, body mass index; aHR, Adjuster hazard ratio; CI, confidence interval; Ref, reference.

Supplemental Table 8. Risk of incident ulcerative colitis according to physical activity and sedentary time stratified by median TDI categories^a^

| Subgroup | Low TDI | | |  | | |  |
| --- | --- | --- | --- | --- | --- | --- | --- |
| Types of physical activity | Total | Events (rateb) | aHR (95%CI) | Total | Events (rate^b^) | aHR (95%CI) |  |
| Light physical activity, min/week | | | |  |  |  | 0.808 |
| Q1 (<1634.9) | 11168 | 21 (2.2) | Ref | 12507 | 45 (4.2) | Ref |  |
| Q2 (1634.9-to<2068.7) | 11890 | 25 (2.4) | 1.22 (0.68, 2.18) | 11778 | 39 (3.8) | 0.93 (0.60, 1.43) |  |
| Q3 (2068.7-to<2552.1) | 12196 | 18 (1.7) | 0.91 (0.48, 1.73) | 11481 | 30 (3.0) | 0.72 (0.45, 1.16) |  |
| Q4 (>2552.1) | 11929 | 28 (2.7) | 1.50 (0.83, 2.71) | 11727 | 37 (3.6) | 0.88 (0.56, 1.39) |  |
| Moderate to vigorous physical activity, min/week | | | |  |  |  | 0.160 |
| Q1 (<112.5) | 11945 | 31 (3.0) | Ref | 11798 | 50 (4.9) | Ref |  |
| Q2 (112.5-to<231.5) | 11916 | 18 (1.7) | 0.59 (0.33, 1.07) | 11681 | 47 (4.6) | 0.97 (0.65, 1.46) |  |
| Q3 (231.5-to<402.3) | 11619 | 21 (2.1) | 0.70 (0.39, 1.24) | 12070 | 25 (2.4) | 0.51 (0.31, 0.84) |  |
| Q4 (>402.3) | 11703 | 22 (2.1) | 0.69 (0.38, 1.24) | 11944 | 29 (2.8) | 0.59 (0.36, 0.96) |  |
| Sedentary time, min/week | | |  |  |  |  | 0.392 |
| Q1 (<3437.9) | 12103 | 26 (2.5) | Ref | 11570 | 32 (3.2) | Ref |  |
| Q2 (3437.9-to<3950.9) | 12236 | 24 (2.2) | 0.90 (0.51, 1.57) | 11436 | 29 (2.9) | 0.92 (0.56, 1.52) |  |
| Q3 (3950.9-to<4457.8) | 11748 | 23 (2.2) | 0.85 (0.48, 1.50) | 11920 | 43 (4.1) | 1.35 (0.85, 2.14) |  |
| Q4 (>4457.8) | 11096 | 19 (2.0) | 0.69 (0.37, 1.28) | 12567 | 47 (4.3) | 1.40 (0.88, 2.22) |  |

^a^ Adjusted for age, sex, body mass index, educational level, smoking status, alcohol consumption, and pre-existing chronic conditions (hypertension, diabetes and cardiovascular disease), non-steroid anti-inflammatory drug use, antibiotics use, and, among female participants, use of oral contraception, menopausal status, hormone therapy and parity.

^b^ Incidence rates per 10000 person-years.

^c^ *P* for interaction, The interaction between TDI and PA categories were exaimed by liklihood ratio test. Specifically, we established two models, one without the inclusion of interaction terms [quartiles of PA (factor), TDI, and all covariates] and the other with the inclusion of interaction terms [quartiles of PA (factor), TDI, quartiles of PA (factor) × TDI, and all covariates]. Likelihood ratio tests were employed to compare the disparities between these two models, if the *P-*value is significant, it suggests a difference between the two models, indicating the potential presence of interaction effects.

TDI, Townsend Deprivation Index; aHR, Adjuster hazard ratio; CI, confidence interval; Ref, reference.

Supplemental Table 9. Risk of incident ulcerative colitis according to physical activity and sedentary time stratified by smoking status categories^a^

| Subgroup | Never Smokers | | |  | | |  |
| --- | --- | --- | --- | --- | --- | --- | --- |
| Types of physical activity | Total | Events (rate^b^) | aHR (95%CI) | Total | Events (rate^b^) | aHR (95%CI) |  |
| Light physical activity, min/week | | | |  |  |  | 0.443 |
| Q1 (<1634.9) | 13203 | 24 (2.1) | Ref | 10472 | 42 (4.7) | Ref |  |
| Q2 (1634.9-to<2068.7) | 13392 | 32 (2.7) | 1.36 (0.80, 2.32) | 10276 | 32 (3.6) | 0.83 (0.52, 1.33) |  |
| Q3 (2068.7-to<2552.1) | 13564 | 24 (2.0) | 1.00 (0.56, 1.79) | 10113 | 24 (2.7) | 0.66 (0.40, 1.10) |  |
| Q4 (>2552.1) | 13913 | 29 (2.4) | 1.16 (0.66, 2.05) | 9743 | 36 (4.2) | 1.04 (0.65, 1.67) |  |
| Moderate to vigorous physical activity, min/week | | | |  |  |  | 0.349 |
| Q1 (<112.5) | 12705 | 33 (3.0) | Ref | 11038 | 48 (5.1) | Ref |  |
| Q2 (112.5-to<231.5) | 13698 | 25 (2.1) | 0.68 (0.40, 1.15) | 9899 | 40 (4.7) | 0.94 (0.61, 1.45) |  |
| Q3 (231.5-to<402.3) | 13971 | 26 (2.1) | 0.68 (0.40, 1.16) | 9718 | 20 (2.4) | 0.48 (0.28, 0.82) |  |
| Q4 (>402.3) | 13698 | 25 (2.1) | 0.63 (0.36, 1.10) | 9949 | 26 (3.0) | 0.60 (0.36, 0.99) |  |
| Sedentary time, min/week | | |  |  |  |  | 0.509 |
| Q1 (<3437.9) | 13921 | 31 (2.5) | Ref | 9752 | 27 (3.2) | Ref |  |
| Q2 (3437.9-to<3950.9) | 13558 | 20 (1.7) | 0.68 (0.39, 1.20) | 10114 | 33 (3.8) | 1.18 (0.71, 1.96) |  |
| Q3 (3950.9-to<4457.8) | 13444 | 30 (2.5) | 1.05 (0.63, 1.74) | 10224 | 36 (4.1) | 1.24 (0.75, 2.06) |  |
| Q4 (>4457.8) | 13149 | 28 (2.4) | 0.99 (0.58, 1.68) | 10514 | 38 (4.2) | 1.18 (0.71, 1.97) |  |

^a^ Adjusted for age, sex, body mass index, Townsend Deprivation Index, educational level, alcohol consumption, and pre-existing chronic conditions (hypertension, diabetes and cardiovascular disease), non-steroid anti-inflammatory drug use, antibiotics use, and, among female participants, use of oral contraception, menopausal status, hormone therapy and parity.

^b^ Incidence rates per 10000 person-years.

^c^ *P* for interaction, The interaction between smoking status and PA categories were exaimed by liklihood ratio test. Specifically, we established two models, one without the inclusion of interaction terms [quartiles of PA (factor), smoking status, and all covariates] and the other with the inclusion of interaction terms [quartiles of PA (factor), smoking status, quartiles of PA (factor) × smoking status, and all covariates]. Likelihood ratio tests were employed to compare the disparities between these two models, if the *P-*value is significant, it suggests a difference between the two models, indicating the potential presence of interaction effects.

AHR, Adjuster hazard ratio; CI, confidence interval; Ref, reference.

Supplemental Table 10. Risk of incident ulcerative colitis according to physical activity and sedentary time stratified by CVD categories^a^

| Types of physical activity | Without CVD | | | With CVD | | | *P* for interaction^c^ |
| --- | --- | --- | --- | --- | --- | --- | --- |
|  | Total | Events (rate^b^) | aHR (95%CI) | Total | Events (rate^b^) | aHR (95%CI) |  |
| Light physical activity, min/week | | | |  |  |  | 0.799 |
| Q1 (<1634.9) | 22306 | 57 (0.3) | Ref | 1365 | 9 (0.8) | Ref |  |
| Q2 (1634.9-to<2068.7) | 22302 | 55 (0.3) | 1.01 (0.69, 1.46) | 1365 | 6 (0.5) | 0.73 (0.26, 2.07) |  |
| Q3 (2068.7-to<2552.1) | 22305 | 49 (0.3) | 0.90 (0.61, 1.33) | 1366 | 4 (0.3) | 0.51 (0.15, 1.70) |  |
| Q4 (>2552.1) | 22303 | 55 (0.3) | 1.00 (0.68, 1.48) | 1364 | 8 (0.7) | 0.96 (0.35, 2.62) |  |
| Moderate to vigorous physical activity, min/week | | | | |  |  | 0.617 |
| Q1 (<112.5) | 22307 | 70 (0.4) | Ref | 1365 | 9 (0.8) | Ref |  |
| Q2 (112.5-to<231.5) | 22351 | 58 (0.3) | 0.82 (0.57, 1.16) | 1366 | 8 (0.7) | 0.97 (0.36, 2.57) |  |
| Q3 (231.5-to<402.3) | 22256 | 41 (0.2) | 0.57 (0.38, 0.84) | 1367 | 7 (0.6) | 0.91 (0.32, 2.56) |  |
| Q4 (>402.3) | 22302 | 47 (0.2) | 0.62 (0.42, 0.92) | 1362 | 3 (0.3) | 0.39 (0.10, 1.52) |  |
| Sedentary time, min/week | | |  |  |  |  | 0.383 |
| Q1 (<3437.9) | 22304 | 54 (0.3) | Ref | 1366 | 5 (0.4) | Ref |  |
| Q2 (3437.9-to<3950.9) | 22305 | 46 (0.2) | 0.86 (0.58, 1.27) | 1364 | 6 (0.5) | 1.35 (0.41, 4.45) |  |
| Q3 (3950.9-to<4457.8) | 22305 | 58 (0.3) | 1.08 (0.75, 1.58) | 1366 | 7 (0.6) | 1.45 (0.45, 4.63) |  |
| Q4 (>4457.8) | 22302 | 58 (0.3) | 1.05 (0.72, 1.55) | 1364 | 9 (0.8) | 1.72 (0.55, 5.33) |  |

^a^ Adjusted for age, sex, body mass index, Townsend Deprivation Index, educational level, smoking status, alcohol consumption, and pre-existing chronic conditions (hypertension and diabetes), non-steroid anti-inflammatory drug use, antibiotics use, and, among female participants, use of oral contraception, menopausal status, hormone therapy and parity.

^b^ Incidence rates per 10000 person-years.

^c^ *P* for interaction, the interaction between CVD and PA categories were exaimed by liklihood ratio test. Specifically, we established two models, one without the inclusion of interaction terms [quartiles of PA (factor), CVD, and all covariates] and the other with the inclusion of interaction terms [quartiles of PA (factor), CVD, quartiles of PA (factor) × CVD, and all covariates]. Likelihood ratio tests were employed to compare the disparities between these two models, if the *P-*value is significant, it suggests a difference between the two models, indicating the potential presence of interaction effects.

CVD, cardiovascular disease; aHR, Adjuster hazard ratio; CI, confidence interval; Ref, reference.

Supplemental Table 11. Sample characteristics stratified at the median activity threshold of ≥231.5 minutes of MVPA per week (sample median)

| Characteristic | Regular activity | Weekend warrior | Inactive |
| --- | --- | --- | --- |
| N | 20832 | 26235 | 47032 |
| Sex |  |  |  |
| Female | 10343 (49.6) | 12260 (46.7) | 30425 (64.7) |
| Male | 10489 (50.4) | 13975 (53.3) | 16607 (35.3) |
| Age at accelerometer assessment | 61.04 (7.88) | 62.14 (7.69) | 62.96 (7.86) |
| BMI (kg/m2) | 25.67 (3.91) | 25.93 (3.80) | 27.61 (4.97) |
| Townsend Index score | -1.34 (3.00) | -1.96 (2.69) | -1.77 (2.79) |
| College degree or equivalent | 10799 (51.8) | 13087 (49.9) | 18097 (38.5) |
| Cigarette smoking |  |  |  |
| Never | 12002 (57.6) | 15482 (59.0) | 26232 (55.8) |
| Previous | 7556 (36.3) | 9296 (35.4) | 16997 (36.1) |
| Current | 1274 (6.1) | 1457 (5.6) | 3803 (8.1) |
| Alcohol consumption |  |  |  |
| never | 1065 (5.1) | 1102 (4.2) | 3148 (6.7) |
| <1 time/week | 3658 (17.6) | 4222 (16.1) | 11268 (24.0) |
| 1-2 time/week | 5030 (24.1) | 6634 (25.3) | 11957 (25.4) |
| 3-4 time/week | 5864 (28.1) | 7722 (29.4) | 10914 (23.2) |
| >4 time/week | 5215 (25.0) | 6555 (25.0) | 9745 (20.7) |
| Pre-existing chronic conditions | 19932 (95.7) | 25028 (95.4) | 43710 (92.9) |
| Cardiovascular disease | 900 (4.3) | 1207 (4.6) | 3322 (7.1) |
| Hypertension | 4624 (22.2) | 6102 (23.3) | 14770 (31.4) |
| Diabetes | 639 (3.1) | 776 (3.0) | 2942 (6.3) |
| Regular use of NSAID* | 4710 (22.6) | 5969 (22.8) | 12829 (27.3) |
| Antibiotic use for last 3 months | 248 (1.2) | 354 (1.3) | 667 (1.4) |
| female-specific variables | 3662 (35.4) | 3680 (30.0) | 9838 (32.3) |
| Postmenopause | 6681 (64.6) | 8580 (70.0) | 20587 (67.7) |
| parity |  |  |  |
| 0 | 2789 (27.0) | 2805 (22.9) | 5798 (19.1) |
| 1-2 | 5527 (53.4) | 6878 (56.1) | 17836 (58.6) |
| 3+ | 2027 (19.6) | 2577 (21.0) | 6791 (22.3) |
| Ever use of oral contraceptives | 8937 (86.4) | 10539 (86.0) | 25777 (84.7) |
| Ever use of HRT | 3273 (31.6) | 4345 (35.4) | 12977 (42.7) |

Continuous variables are presented as mean (SD); categorical variables are presented as n (%).

^a^ Townsend deprivation index = a composite area-level measure of deprivation based on unemployment, non-car ownership, non-home ownership, and household overcrowding; a higher score indicates higher deprivation.

^b^ Regular NSAID use was assessed through a questionnaire. Participants were asked about their regular use (defined as most days of the week for the last 4 weeks) of common over-the-counter medications, including various brand names for aspirin, ibuprofen, paracetamol, and codeine. The questionnaire provided a comprehensive list of brand names to ensure accurate reporting of NSAID use, including combinations and less commonly recognized trade names.

MVPA, moderate-to-vigorous physical activity; BMI, Body Mass Index, NSAID, nonsteroidal anti-inflammatory drug, HRT, hormone replacement therapy

Supplemental Table 12. Sample characteristics stratified at the median activity threshold of ≥150 minutes of MVPA per week (guideline-based threshold)

| Characteristic | Regular activity | Weekend warrior | Inactive |
| --- | --- | --- | --- |
| N | 23913 | 38772 | 31414 |
| Sex |  |  |  |
| Female | 12256 (51.3) | 19797 (51.1) | 20975 (66.8) |
| Male | 11657 (48.7) | 18975 (48.9) | 10439 (33.2) |
| Age at accelerometer assessment | 61.16 (7.9) | 62.26 (7.7) | 63.23 (7.8) |
| BMI (kg/m2) | 25.80 (4.0) | 26.17 (4.0) | 28.07 (5.2) |
| Townsend Index score^a^ | -1.33 (3.0) | -1.94 (2.7) | -1.77 (2.8) |
| College degree or equivalent | 12133 (50.7) | 18561 (47.9) | 11289 (35.9) |
| Cigarette smoking |  |  |  |
| Never | 13777 (57.6) | 22816 (58.8) | 17123 (54.5) |
| Previous | 8626 (36.1) | 13734 (35.4) | 11489 (36.6) |
| Current | 1510 (6.3) | 2222 (5.7) | 2802 (8.9) |
| Alcohol consumption |  |  |  |
| never | 1258 (5.3) | 1741 (4.5) | 2316 (7.4) |
| <1 time/week | 4404 (18.4) | 6746 (17.4) | 7998 (25.5) |
| 1-2 time/week | 5749 (24.0) | 9950 (25.7) | 7922 (25.2) |
| 3-4 time/week | 6610 (27.6) | 10927 (28.2) | 6963 (22.2) |
| >4 time/week | 5892 (24.6) | 9408 (24.3) | 6215 (19.8) |
| Pre-existing chronic conditions | 22840 (95.5) | 36871 (95.1) | 28959 (92.2) |
| Cardiovascular disease | 1073 (4.5) | 1901 (4.9) | 2455 (7.8) |
| Hypertension | 5453 (22.8) | 9493 (24.5) | 10550 (33.6) |
| Diabetes | 791 (3.3) | 1254 (3.2) | 2312 (7.4) |
| Regular use of NSAID^b^ | 5490 (23.0) | 9172 (23.7) | 8846 (28.2) |
| Antibiotic use for last 3 months | 284 (1.2) | 540 (1.4) | 445 (1.4) |
| female-specific variables | 4326 (35.3) | 6059 (30.6) | 6795 (32.4) |
| Postmenopause | 7930 (64.7) | 13738 (69.4) | 14180 (67.6) |
| parity |  |  |  |
| 0 | 3220 (26.3) | 4250 (21.5) | 3922 (18.7) |
| 1-2 | 6623 (54.0) | 11300 (57.1) | 12318 (58.7) |
| 3+ | 2413 (19.7) | 4247 (21.5) | 4735 (22.6) |
| Ever use of oral contraceptives | 10562 (86.2) | 16981 (85.8) | 17710 (84.4) |
| Ever use of HRT | 4023 (32.8) | 7315 (37.0) | 9257 (44.1) |

Continuous variables are presented as mean (SD); categorical variables are presented as n (%).

^a^ Townsend deprivation index = a composite area-level measure of deprivation based on unemployment, non-car ownership, non-home ownership, and household overcrowding; a higher score indicates higher deprivation.

^b^ Regular NSAID use was assessed through a questionnaire. Participants were asked about their regular use (defined as most days of the week for the last 4 weeks) of common over-the-counter medications, including various brand names for aspirin, ibuprofen, paracetamol, and codeine. The questionnaire provided a comprehensive list of brand names to ensure accurate reporting of NSAID use, including combinations and less commonly recognized trade names.

MVPA, moderate-to-vigorous physical activity; BMI, Body Mass Index, NSAID, nonsteroidal anti-inflammatory drug, HRT, hormone replacement therapy

Supplemental Table 13. Associations between physical activity pattern and incident ulcerative colitis with 2-year blanking period after accelerometer^a^

| Activity threshold | Activity Pattern^b^ | Event (rate^c^) | Hazard ratio  (95% CI) | *p*_value |
| --- | --- | --- | --- | --- |
| 25th percentile (≥112.5 minutes MVPA/week) | Inactive (n=23,480) | 65 (3.22) | Ref | Ref |
|  | Weekend warrior (45559) | 78 (1.96) | 0.65 (0.46-0.92) | 0.015 |
|  | Regular activity (25060) | 42 (1.91) | 0.60 (0.40-0.91) | 0.016 |
| Guideline-based (≥150 minutes MVPA/week) | Inactive (n=31,414) | 81 (2.99) | Ref | Ref |
|  | Weekend warrior (38772) | 64 (1.89) | 0.67 (0.48-0.95) | 0.023 |
|  | Regular activity (23913) | 40 (1.91) | 0.64 (0.43-0.96) | 0.029 |
| 50th percentile (≥231.5 minutes MVPA/week) | Inactive (n=47,032) | 114 (2.80) | Ref | Ref |
|  | Weekend warrior (26235) | 39 (1.70) | 0.64 (0.44-0.93) | 0.018 |
|  | Regular activity (20832) | 32 (1.75) | 0.62 (0.41-0.93) | 0.021 |
| 75th percentile (≥402.0 minutes MVPA/week) | Inactive (n=70,568) | 148 (2.41) | Ref | Ref |
|  | Weekend warrior (11044) | 18 (1.86) | 0.82 (0.50-1.35) | 0.432 |
|  | Regular activity (12487) | 19 (1.73) | 0.72 (0.44-1.17) | 0.188 |

^a^ Adjusted for age, sex, body mass index, Townsend Deprivation Index, educational level, smoking status, alcohol consumption, and pre-existing chronic conditions (hypertension, diabetes and cardiovascular disease), non-steroid anti-inflammatory drug use, antibiotics use, and, among female participants, use of oral contraception, menopausal status, hormone therapy and parity.

^b^ Inactive defined as weekly MVPA below the specified threshold.

^c^ Incidence rates per 10000 person-years.

MVPA, moderate-to-vigorous physical activity; CI, confidence interval; Ref, reference.

Supplemental Table 14 Associations between physical activity pattern and incident ulcerative colitis across varying MVPA threshold based on competitive risk model (Fine and Gray)^a^

| Activity threshold | Activity Pattern^b^ | Event (rate^c^) | Death | SHR  (95% CI) | *p*_value |
| --- | --- | --- | --- | --- | --- |
| 25th percentile (≥112.5 minutes MVPA/week) | Inactive (n=23,480) | 81 (4.01) | 1503 | Ref | Ref |
|  | Weekend warrior (45559) | 111(2.78) | 1602 | 0.75 (0.56-0.99) | 0.045 |
|  | Regular activity (25060) | 51 (2.32) | 864 | 0.59 (0.41-0.85) | 0.005 |
| Guideline-based (≥150 minutes MVPA/week) | Inactive (n=31,414) | 101 (3.73) | 1842 | Ref | Ref |
|  | Weekend warrior (38772) | 93 (2.74) | 1314 | 0.78 (0.59-1.04) | 0.096 |
|  | Regular activity (23913) | 49 (2.34) | 813 | 0.64 (0.45-0.91) | 0.013 |
| 50th percentile (≥231.5 minutes MVPA/week) | Inactive (n=47,032) | 147 (3.61) | 2438 | Ref | Ref |
|  | Weekend warrior (26235) | 58 (2.52) | 857 | 0.73 (0.53-1.00) | 0.049 |
|  | Regular activity (20832) | 38 (2.08) | 674 | 0.57 (0.39-0.83) | 0.003 |
| 75th percentile (≥402.0 minutes MVPA/week) | Inactive (n=70,568) | 194 (3.16) | 3284 | Ref | Ref |
|  | Weekend warrior (11044) | 25 (2.58) | 342 | 0.86 (0.56-1.31) | 0.47 |
|  | Regular activity (12487) | 24 (2.18) | 342 | 0.69 (0.45-1.07) | 0.096 |

^a^ Adjusted for age, sex, body mass index, Townsend Deprivation Index, educational level, smoking status, alcohol consumption, and pre-existing chronic conditions (hypertension, diabetes and cardiovascular disease), non-steroid anti-inflammatory drug use, antibiotics use, and, among female participants, use of oral contraception, menopausal status, hormone therapy and parity.

^b^ Inactive defined as weekly MVPA below the specified threshold.

^c^ Incidence rates per 10000 person-years.

MVPA, moderate-to-vigorous physical activity; SHR, subdistribution hazard ratio; CI, confidence interval; Ref, reference.

**Supplemental Figure**

Supplemental Figure 1 Weekly MVPA distribution: Top two days vs remaining five days in active individuals (active: ≥150 min/week MVPA, guideline-based threshold)

MVPA distribution for active individuals (≥150 min/week, n=61,965): 2 most active days (green) vs remaining 5 (yellow). A: Weekend warriors (≥50% MVPA in 1-2 days). B: Regular actives. Note: 491 weekend warriors (1.3%) with zero MVPA on 5 days assigned 1 min for log scale. MVPA indicates moderate to vigorous physical activity.
